# The Multiple Promotion Effects of Ammonium Phosphate-Modified Ag_3_PO_4_ on Photocatalytic Performance

**DOI:** 10.3389/fchem.2019.00866

**Published:** 2019-12-24

**Authors:** Qin Liu, Na Li, Zheng Qiao, Wenjuan Li, Linlin Wang, Shuao Zhu, Zhihong Jing, Tingjiang Yan

**Affiliations:** ^1^The Key Laboratory of Life-Organic Analysis, College of Chemistry and Chemical Engineering, Qufu, China; ^2^Qufu Normal University Library, Qufu Normal University, Qufu, China

**Keywords:** ammonium phosphate, surface modification, Ag_3_PO_4_, metallic Ag, reactive species

## Abstract

Phosphate (${\text{PO}}_{4}^{3-}$) modification of semiconductor photocatalysts such as TiO_2_, C_3_N_4_, BiVO_4_, and etc. has been shown positive effect on the enhancement of photocatalytic performance. In the present study, we demonstrate a novel one-pot surface modification route on Ag_3_PO_4_ photocatalyst by ammonium phosphate [(NH_4_)_3_PO_4_], which combines ${\text{PO}}_{4}^{3-}$ modification with ammonium (${\text{NH}}_{4}^{+}$) etching to show multiple effects on the structural variation of Ag_3_PO_4_ samples. The modified Ag_3_PO_4_ photocatalysts exhibit much higher photocatalytic performance than bare Ag_3_PO_4_ for the degradation of organic dye solutions under visible light irradiation. It is indicated that the ${\text{NH}}_{4}^{+}$ etching favors the surface transition from Ag_3_PO_4_ to metallic Ag nanoparticles, resulting in the fast capture of photogenerated electrons and the followed generation of ${\text{O}}_{2}^{\cdot \text{−}}$ radicals. The strongly adsorbed ${\text{PO}}_{4}^{3-}$ on the Ag_3_PO_4_ surfaces can further provide more negative electrostatic field, which improves the separation of photogenerated electron-hole pairs by inducing the holes to directly flow to the surface and then enhances the formation of reactive **·**OH radicals. Furthermore, the photocatalytic performance of the modified Ag_3_PO_4_ photocatalysts can be optimized by monitoring the concentration of (NH_4_)_3_PO_4_ that is 1 mM.

## Introduction

In recent years, photocatalytic technology has received widespread attention in wastewater treatment and energy development. At present, although titanium dioxide is the most widely used photocatalyst, the wide band energy, and the recombination of the photogenerated electron-hole limit its application (Asahi et al., 2001; Yan et al., 2014a; Qi et al., 2016, 2018). Many new semiconductor materials have then developed in recent years, such as ZnO (Qi et al., 2017), CdS (Jing and Guo, 2006; Dai et al., 2018), WO_3_ (Liu et al., 2019), Ag_2_WO_4_ (Macedo et al., 2018), BiVO_4_ (Wang et al., 2018, 2019; Song et al., 2019), AgCl (Han et al., 2011), C_3_N_4_ (Guo et al., 2019; Huo et al., 2019; Qi et al., 2019), etc. In 2010, Yi et al. (2010) reported that Ag_3_PO_4_ has noticeable absorption in the UV-visible spectral range, which can utilize visible light to oxidize water to produce oxygen and degrade organic contaminants to purify water resources. However, the photo-corrosion phenomenon and the low photocatalytic efficiency due to the fast recombination of photogenerated carriers restrict the wide application of Ag_3_PO_4_ (Martin et al., 2015). Accordingly, several attempts have been proposed to enhance its photocatalytic activity and improve the photostability with some success, such as morphology and/or size control (Dong et al., 2013, 2014; Li et al., 2014; Krungchanuchat et al., 2017), metal deposition (Liu et al., 2012; Yan et al., 2014b; Lin et al., 2019), coupling with other semiconductors to form Z-scheme heterostructures (Chen et al., 2017; Wang et al., 2017; Li et al., 2019; Zhang et al., 2019).

Surface modification can be an alternative route to boost the photocatalytic performance by changing the charge transfer pathways that typically take place at the surfaces of photocatalysts (Zhao et al., 2008; Li et al., 2015). Many researchers have been reported that anions such as F^−^, ${\text{PO}}_{4}^{3-}$, and ${\text{SO}}_{4}^{2-}$ can greatly change the interfacial and surface chemistry of pristine photocatalysts like TiO_2_, BiPO_4_, Fe_2_O_3_, C_3_N_4_, etc. and enhance the photocatalytic performance (Park and Choi, 2004; Mohapatra and Parida, 2006; Kim and Choi, 2007; Korosi et al., 2007; Parida et al., 2008). Among them, ${\text{PO}}_{4}^{3-}$ anions (phosphate) are known to exhibit a strong ability to adsorb onto the surfaces of semiconductor photocatalysts by substituting surface hydroxyl groups. Jing et al. have demonstrated that the photocatalytic activity for water oxidation over phosphate-modified TiO_2_ was notably improved because the negative charges on the TiO_2_ surface resulting from the phosphate groups (–Ti–O–P–O^−^) promoted the charge separation (Jing et al., 2012a,b). The surface phosphate modification can also significantly enhance the reactive oxygen species and therefore prolong the photogenerated charge carrier lifetime and improve the separation efficiency (Liu et al., 2014; Min et al., 2014). For instance, Li and co-workers have demonstrated that the surface hydroxyl concentration of the phosphate-modified BiPO_4_ samples is increased and responsible for the generation of more hydroxyl radicals to participate in the methylene orange (MO) degradation. As the aforementioned, Ag_3_PO_4_ photocatalyst suffers from serious photo corrosion issues due to the reduction of Ag_3_PO_4_ into metallic Ag by photogenerated electrons. The metallic Ag nanoparticles can also be formed by the reaction of Ag^+^ in Ag_3_PO_4_ with the thermally excited electrons along with the creation of structural defects (oxygen and/or silver vacancies) during the thermal annealing process (Yan et al., 2016). Our recent work further showed that the composition and morphology of Ag_3_PO_4_ can be tuned using ammonia solution etching, which mentions that the strong interaction between surface Ag atoms and ammonia aroused that the surface Ag atoms spontaneous dissolution, resulting in the face-selective etching over Ag_3_PO_4_ dodecahedron and the formation of Ag/Ag_3_PO_4_ photocatalyst (Zhai et al., 2016). Inspiration by the promotion effect of surface modification on the reported photocatalysts and the structural instability of Ag_3_PO_4_, it is expected that the simultaneous modification by phosphate and ammonia etching on Ag_3_PO_4_ could alter its structure and induce positive effects on the photogenerated charge separation and the reactive species.

In this work, we developed a one-pot surface modification route by using ammonium phosphate [(NH_4_)_3_PO_4_] to achieve the multiple promotion effects on structural variation and photocatalytic performance of Ag_3_PO_4_. The pristine Ag_3_PO_4_ was synthesized by the precipitation method and modified by a general immersion process in different concentrations of (NH_4_)_3_PO_4_ solutions, followed by a thermal annealing process. The chemical etching occurs on the surface of Ag_3_PO_4_ and induces the formation of Ag^0^ nanoparticles due to the strong coordination interaction between Ag^+^ and ${\text{NH}}_{4}^{+}$ ion, which can act as electrons acceptors to promote the separation of charge carriers and favor the formation of reactive ${\text{O}}_{2}^{\cdot \text{−}}$ species. Meanwhile, the enrichment in the negative electrostatic field formed by the surface bounded ${\text{PO}}_{4}^{3-}$ is favorable for the selective adsorption of cationic dyes, the fast transfer of holes to surfaces and the formation of **·**OH radicals. Accordingly, the multiple effect of surface modification of Ag_3_PO_4_ by (NH_4_)_3_PO_4_ contributes to the enhanced photocatalytic activity and stability toward organic dye solutions.

## Experimental

### Preparation of Ag_3_PO_4_ and Ammonium Phosphate-Modified Ag_3_PO_4_ Samples

All the involved chemicals were purchased from the Shanghai reagent company and used without further purification. Pure Ag_3_PO_4_ was prepared by the reported precipitation method at room temperature (Yan et al., 2014c). 0.3 g of the as-prepared Ag_3_PO_4_ samples were put into the aqueous ammonium phosphate solution (50 mL) with different concentrations (0.5 mM, 1 mM, 5 mM, 10 mM), and the suspension was stirred for 5 h to allow the adsorption and chemical modification on the surface. The ammonium phosphate-modified Ag_3_PO_4_ samples were collected by centrifugation and dried in an oven at 60°C, followed by the thermal annealing in air at 300°C for 3 h in a muffle furnace. The corresponding products were denoted as 0.5P-AP, 1P-AP, 5P-AP, and 10P-AP, respectively. As a reference, the bare Ag_3_PO_4_ was also annealed in air at 300°C for 3 h and denoted as AP.

### Characterizations

X–ray diffraction patterns (XRD) were collected on a Rigaku MinFlex II equipped with Cu K irradiation (λ = 0.15406 nm). Raman spectra of the samples were recorded on a Renishaw Invia Raman microscope. The morphology of the samples was investigated with a field emission scanning electron microscope (FE-SEM) (JEM-2100). X-ray photoelectron spectroscopy (XPS) analysis was conducted on an ESCALAB 250 photoelectron spectroscopy (Thermo Fisher Scientific) at 3.0 × 10^10^ mbar with monochromatic Al K radiation (E = 1,486.2 eV). Fourier transform infrared spectroscopy (FTIR) analysis was carried out by a Nicolet NeXUS 470. UV–visible diffuse reflectance spectra (DRS) of the powders were performed on a Cary 500 Scan Spectrophotometer (Varian, USA) over a range of 200–800 nm, with BaSO_4_ as a reflectance standard. The Brunauer-Emmett-Teller (BET) surface area test was performed on an Auto Chem II surface area analyzer. The charge on the surface (Zeta) of the sample particles in the aqueous solution (pH = 7) is determined by a Nano ZS ZEN3600-type particle size analyzer. The photoluminescence (PL) spectra were obtained by using an F-4600 Fluorescence spectrophotometer with an excitation wavelength of 380 nm. Photoelectrochemical measurements were measured using the Chi660D electrochemical work station and a 300 W Xe lamp equipped with cutoff filters (400 nm <λ <800 nm) as light source. The photocurrent response was detected on an electrochemical workstation (CHI660E, China) using a standard three-electrode cell with a working electrode (as-prepared photocatalyst), a platinum wire as a counter electrode, and an AgCl electrode as a reference electrode in Na_2_SO_4_ solution (0.1 M). All electrochemical potentials are reported vs. NHE.

### Photocatalytic Activity Test

Photocatalytic process were executed in an aqueous solution at room temperature. A 300W Xe lamp equipped with cutoff filters (400 nm < λ < 800 nm) was employed as the irradiation source. Typically, 80 mg specimen as the photocatalyst was dispersed into 80 mL of methyl orange (MO) solution (10 ppm). The suspension was kept stirring in dark condition for 30 min by a blender to establish an adsorption-desorption equilibrium between photocatalyst and MO molecules. Three milliliter of the suspension sample was taken at regular intervals during the process of irradiation and remove the photocatalysts by centrifugation. The residual concentration of the MO dye was detected by the UV-Vis spectrophotometer. The degradation rate is expressed as C/C_0_, where C_0_ is the initial concentration of dye, and C represents the corresponding concentration at a certain time interval. The photocatalytic performance of bare Ag_3_PO_4_ and modified Ag_3_PO_4_ was also estimated by the decomposing dye of rhodamine B (RhB) and methylene blue (MB) under the same condition. Stability is an important and essential property of the photocatalyst, after each catalytic reaction, the final suspension was centrifuged and the solids photocatalyst obtained by centrifugation were washed by water several times and dried at 60°C to obtain a regenerated catalyst which was used to catalyze a new dye solution under the same photocatalytic process.

## Results and Discussion

**Figure 1:**
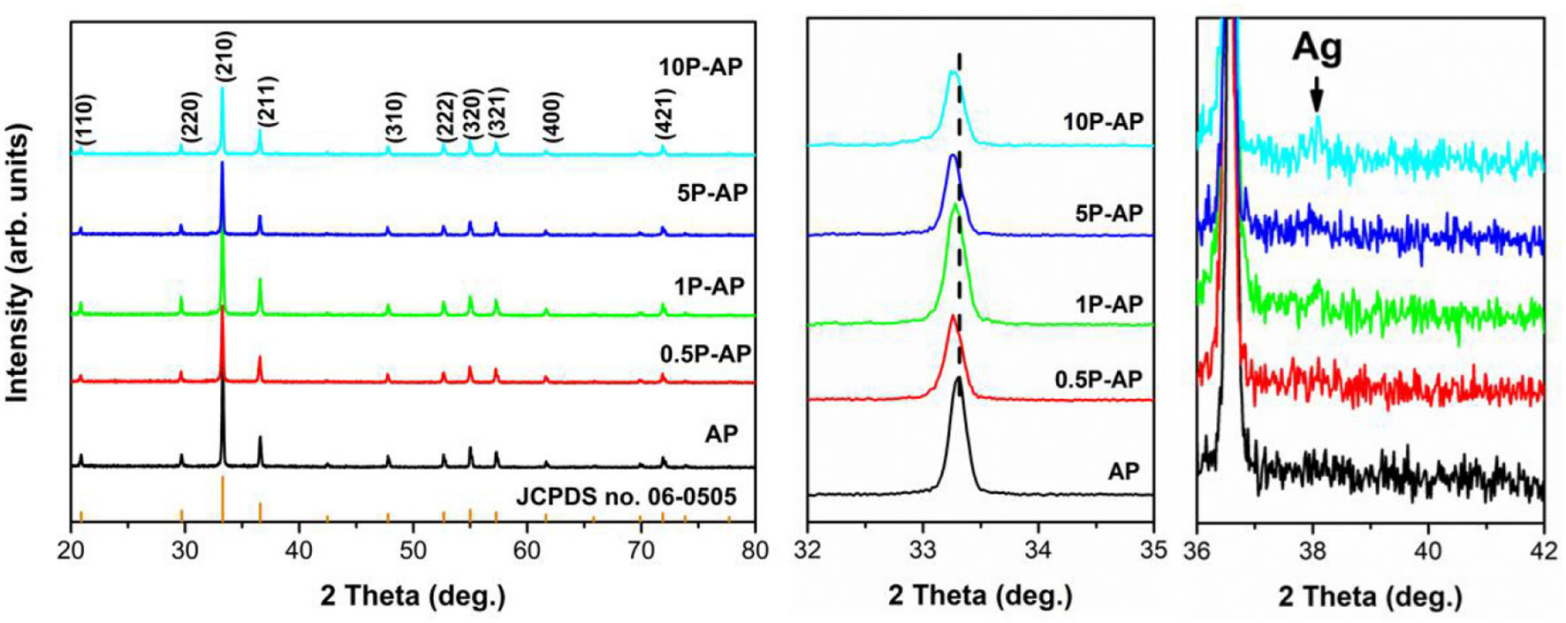
XRD patterns of Ag_3_PO_4_ and ammonium phosphate-modified Ag_3_PO_4_ samples.

Figure 1 shows the XRD patterns of the as-prepared Ag_3_PO_4_ and the ammonium phosphate-modified Ag_3_PO_4_ samples. All the XRD patterns of bare Ag_3_PO_4_ (AP) can be readily indexed to body-centered cubic structure Ag_3_PO_4_ (JCPDS no. 06-0505) (Zhang et al., 2014). The intense and sharp XRD diffraction peaks suggest the bare Ag_3_PO_4_ is highly crystallized. Upon surface modification with ammonium phosphate, the main cubic structure of the modified samples has remained. However, as compared to bare AP, the diffraction peaks of the modified samples show a gradual left shift with increasing the concentration of ammonium phosphate solution. Our previous studies have shown that the surface Ag atoms are easily dissolved from the silver-contained compound after ammonia etching, due to the strong coordination interaction between Ag^+^ and NH_3_ driving the formation of $\text{Ag}({\text{NH}}_{3}{)}_{2}^{+}$ complex ions (Zhai et al., 2016), which would arouse the structural condensation and reset of Ag_3_PO_4_ cells and thereby the separation of interior Ag atoms out from the Ag_3_PO_4_ supercell, responsible to the formation of metallic Ag nanoparticles. In the present study, it is noted that a new diffraction peak appeared at 38.1° in the modified samples (1P-AP, 5P-AP, and 10P-AP) which assigned to (111) plane of Ag^0^ (JCPDS no. 65-2871). Thus, ammonium phosphate can function as a chemical etchant in favoring the formation of new Ag/Ag_3_PO_4_ solid surfaces with distinct structure and composition for Ag_3_PO_4_. The structural variation of Ag_3_PO_4_ upon ammonium phosphate modification is further investigated by Raman spectra (Figure S1). For bare AP, the weak peak at 71 cm^−1^ can be attributed to the external translational and rotational modes associated with the [PO_4_] group (Costa et al., 2018), while the strong peak at 909 cm^−1^ is assigned to the ${\text{PO}}_{4}^{3-}$ symmetric stretching vibration (Sharma et al., 2017). The intensity of these two peaks shows obvious increase after ammonium phosphate modification especially at high concentration, suggesting the strong chemical interaction between ammonium phosphate and Ag_3_PO_4_ particles.

**Figure 2:**
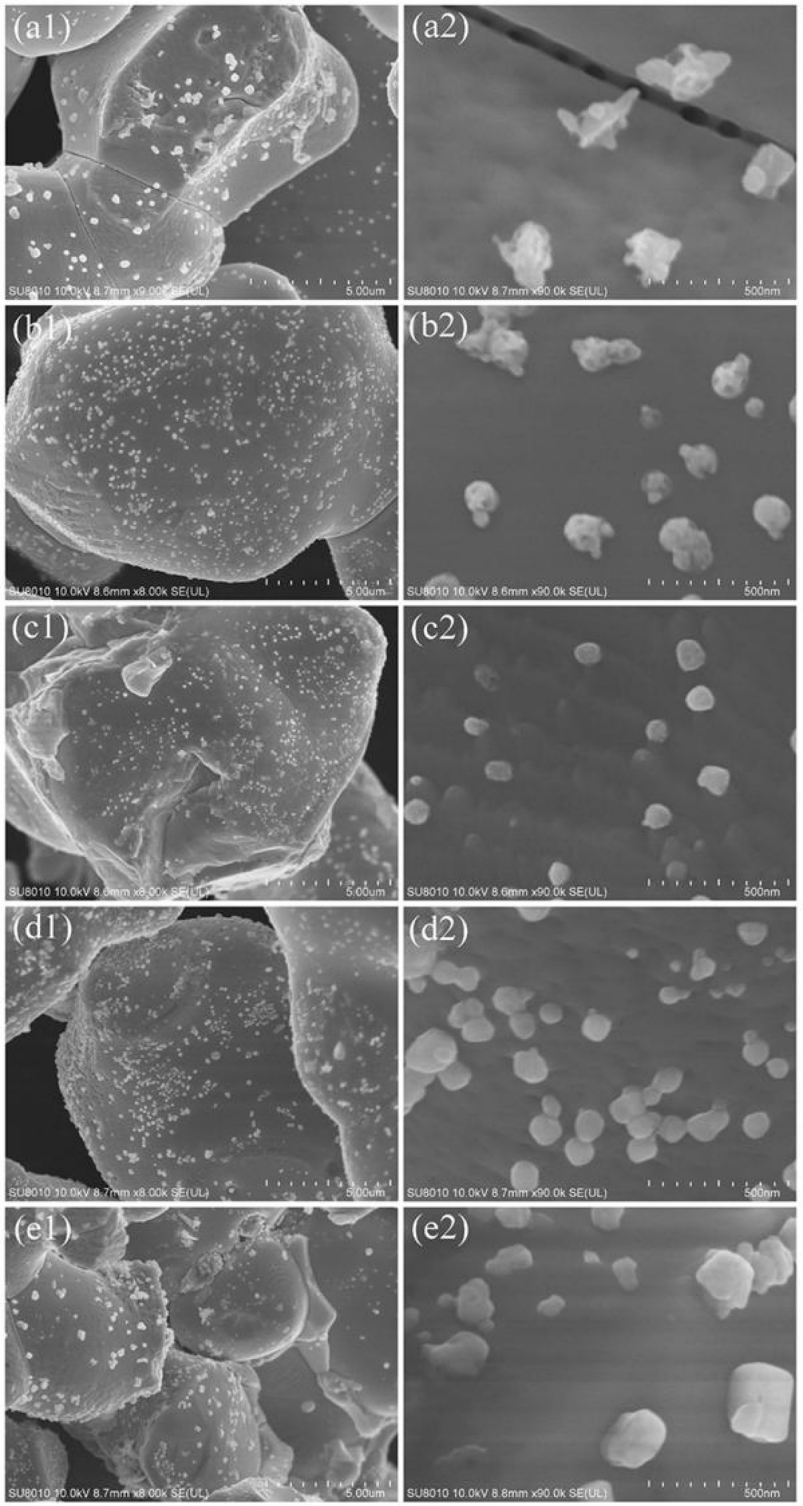
SEM images of bare Ag_3_PO_4_ **(a1,a2)** and ammonium phosphate-modified Ag_3_PO_4_ samples: 0.5P-AP **(b1,b2)**, 1P-AP **(c1,c2)**, 5P-AP **(d1,d2)**, and 10P-AP **(e1,e2)**.

The morphology of the Ag_3_PO_4_ samples before and after surface modification was investigated by SEM (Figure 2). We can observe that the bare Ag_3_PO_4_ is assembled from a plurality of irregular sphere-like particles with obvious fracture surface and several micrometers in size. Meanwhile, many inter-crossed aggregates with a size of ca. 150 nm are observed on the smooth surfaces of sintered Ag_3_PO_4_ particles. These aggregates can be attributed to the formation of Ag^0^ by thermal decomposition of Ag_3_PO_4_ during thermal annealing (Yan et al., 2016, 2017). As for the modified Ag_3_PO_4_ samples, it is obvious that the morphology and the smooth surface of Ag_3_PO_4_ crystals are kept original while the size and distribution of Ag nanoparticles are quite different. When the concentration of ammonium phosphate is 0.5 mM, the size of the Ag nanoparticles decreases markedly and their distribution improves greatly as compared to that on bare Ag_3_PO_4_. This should be due to the strong mutual effect between ${\text{NH}}_{4}^{+}$ and Ag^+^ on the surface and the continuous out-diffusion of Ag nanoparticles. With increasing the concentration of ammonium phosphate to 1 mM, the chemical etching of surface Ag^+^ is proceeded, resulting in the formation of nearly monodispersed Ag nanoparticles which have a diameter around 60 nm instead of aggregates on the Ag_3_PO_4_ surfaces. The higher concentration of ammonium phosphate (5 and 10 mM) further promotes the formation of Ag nanoparticles but with particle growth into ca. 200 nm. The surface modification results in a slight decrease in BET surface area for the as-obtained Ag_3_PO_4_ samples (for example, 3.6 m^2^ g^−1^ for AP and 3.0 m^2^ g^−1^ for 1P-AP) (Table S1) which might be due to the cover of Ag^0^ nanoparticles on the surface of Ag_3_PO_4_ crystals.

**Figure 3:**
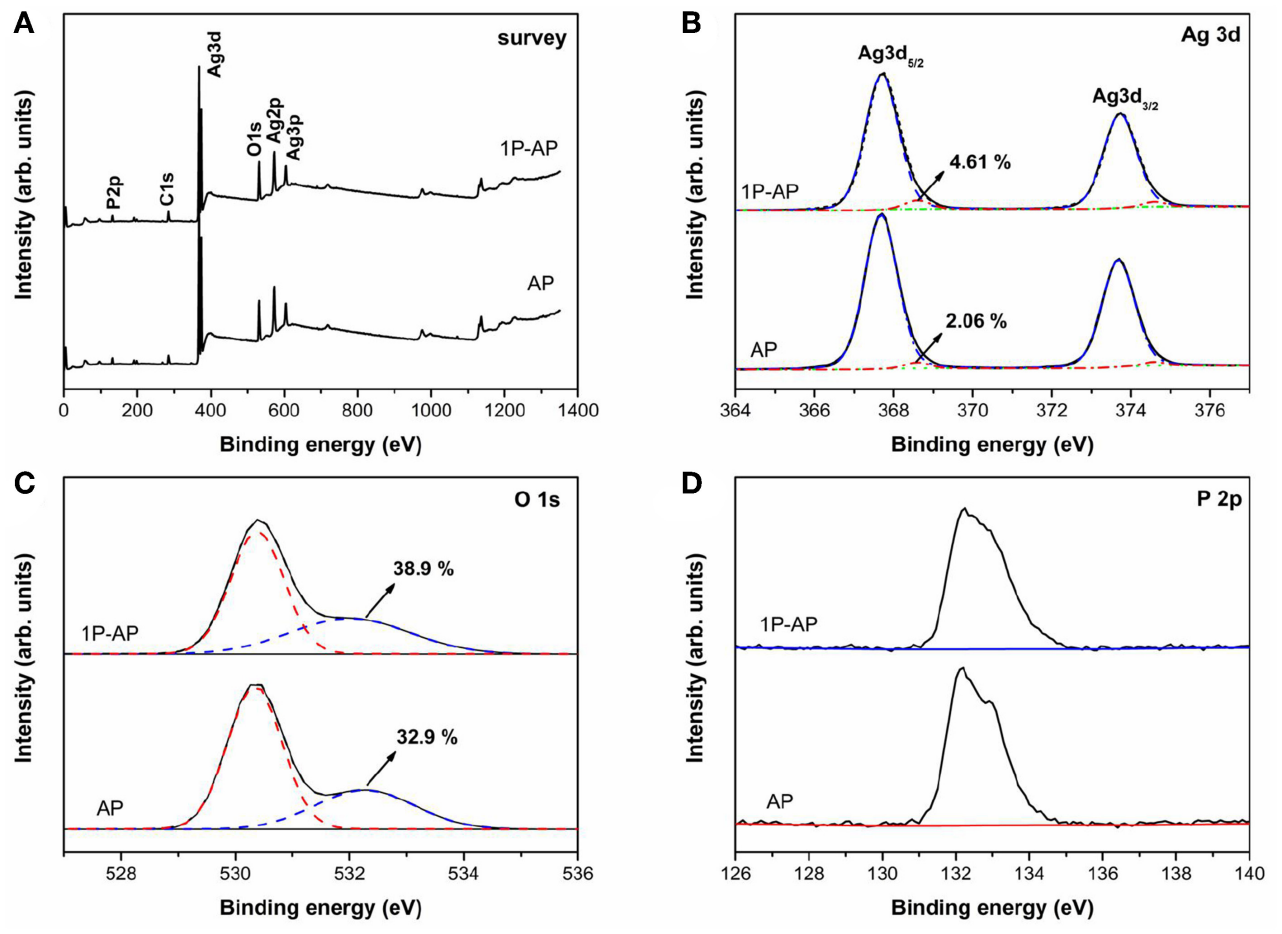
XPS spectra of the as-prepared AP and 1P-AP: **(A)** Survey, **(B)** Ag 3d, **(C)** O 1s, and **(D)** P 2p.

XPS spectra were shown in Figure 3 which invested the surface compositions and chemical states of Ag_3_PO_4_ after modified by ammonium phosphate. Figure 3A show that the samples before and after ammonium phosphate modification are mainly composed of Ag, O, and P elements. The high-resolution XPS spectrum of Ag3d (Figure 3B) indicates two characteristic peaks corresponding to Ag 3d_5/2_ and Ag 3d_3/2_. The peaks of Ag 3d_3/2_ and Ag 3d_5/2_ can be further divided into two different peaks at 374.6, 374.08 eV and 368.6, 368.05 eV, respectively. The peaks at 374.6 and 368.6 eV can be ascribed Ag^0^, while the peaks at 374.08 and 368.05 eV are associated with Ag^+^ ions (Ma et al., 2014; Mao et al., 2018). The calculated percentage composition of Ag^0^ for AP and 1P-AP samples is 2.06 and 4.61%, respectively, which indicates that surface modification promotes the decomposition of Ag_3_PO_4_ into metallic Ag, in good agreement with the XRD and SEM results. The O 1s core level XPS spectra (Figure 3C) could be matched into two peaks at 530.6 and 532.2 eV, which can be assigned to oxides (${\text{O}}_{2}^{-}$) and hydroxyl groups (OH), respectively (Dai et al., 2011; Teng et al., 2013). It is noted that the concentration of surface OH of 1P-AP sample increases significantly after surface modification, which could be attributed to the strong dissociation of H_2_O and binding affinity of phosphate on the Ag_3_PO_4_ surface (Chong et al., 2016). The P 2p in both samples (Figure 3D) is located at 132.5 eV, confirming the valence state of P^5+^ in ${\text{PO}}_{4}^{3-}$ (Wang et al., 2013; Zhang et al., 2019). From the XPS results, we can determine the actual content of (NH_4_)_3_PO_4_ in the 10P-AP sample to be 10.1%, quite consistent with the theoretical value (10.0%) (Table S2). All these results prove that ammonium phosphate modification promotes the formation of Ag/Ag_3_PO_4_ heterostructures and the strong adsorption of ${\text{NH}}_{4}^{+}$ and ${\text{PO}}_{4}^{3-}$ species on the surface of the samples.

**Figure 4:**
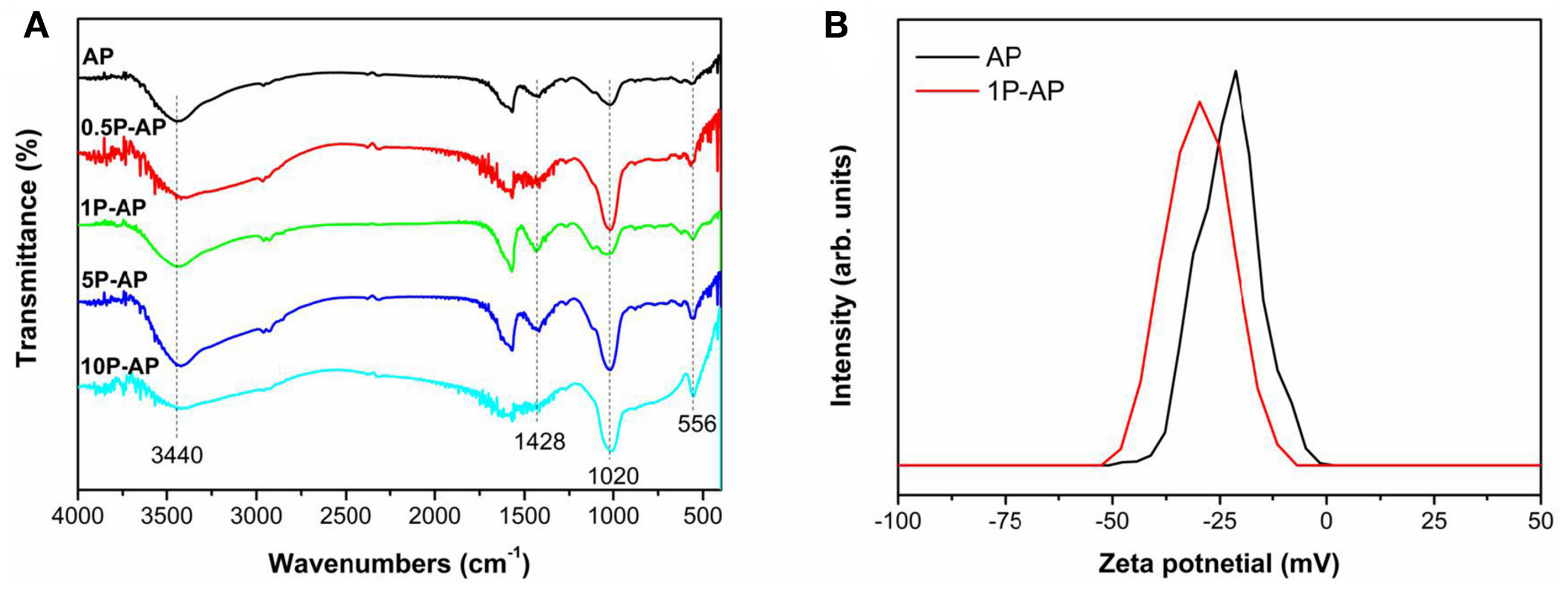
**(A)** FTIR spectra and **(B)** Zeta potential of bare Ag_3_PO_4_ and ammonium phosphate-modified Ag_3_PO_4_ samples.

The strong interaction of phosphate on Ag_3_PO_4_ surface can be supported by FTIR. As shown in Figure 4A, the vibration peaks at 556 and 1,020 cm^−1^ can be assigned to the asymmetrical and symmetrical stretching of ${\text{PO}}_{4}^{3-}$ (Xie et al., 2015; Cruz et al., 2019) while a broad absorption band centered at 1,428 cm^−1^ is assigned to the synergistic effect of P-O stretching vibration and ${\text{PO}}_{4}^{3-}$ symmetric stretching vibrations (Liang et al., 2012). The intensity of all these peaks is gradually enhanced by increasing the concentration of ammonium phosphate, suggesting the strong binding affinity of ${\text{PO}}_{4}^{3-}$ to the Ag_3_PO_4_ surfaces. Moreover, the peak at 3,440 cm^−1^ that is related to the hydroxyl stretching vibration is also enhanced upon surface modification. However, when the concentration of ammonium phosphate is too high to 10 mM, the hydroxyl stretching vibration shows a slight decrease in intensity, which might be because more ${\text{PO}}_{4}^{3-}$ are strongly adsorbed onto the surfaces of Ag_3_PO_4_ by substituting surface hydroxyl groups (Xie et al., 2015). As expected, the strong binding affinity of ${\text{PO}}_{4}^{3-}$ and hydroxyl groups to the Ag_3_PO_4_ surfaces would induce the surface negative electrostatic filed of the as-obtained samples. Figure 4B depicts the Zeta potential of AP and 1P-AP samples in solutions at pH = 7. It is obvious that the Zeta potential for 1P-AP is −29.78 mV, more negative than that of bare AP (−21.34 mV). The increased surface-carried negative charge of Ag_3_PO_4_ after surface modification may improve the selective adsorption of cationic dye and accelerate the migrate of photogenerated holes to the surface, responsible to the obvious enhancement of photocatalytic activity.

**Figure 5:**
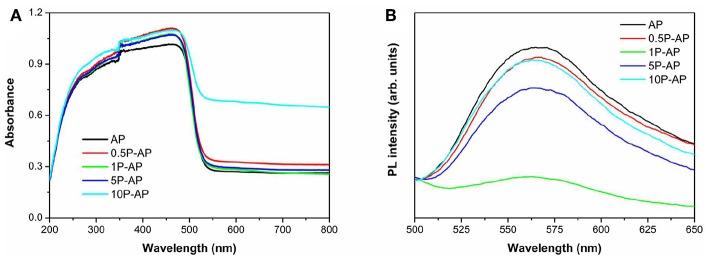
**(A)** UV-Vis diffuse reflectance spectra and **(B)** room temperature PL spectra of bare Ag_3_PO_4_ and ammonium phosphate-modified Ag_3_PO_4_ samples.

The UV-Vis DRS spectra of bare Ag_3_PO_4_ and the modified Ag_3_PO_4_ samples are shown in Figure 5A. We can observe that the bare Ag_3_PO_4_ exhibits the broad solar light absorption in the wavelength range of <530 nm, and the corresponding band gap energy is 2.48 eV. Surface modification has slightly enhanced the absorption in the UV-Vis spectral range but does not induce the change of band gap energy as well as the sample color (Figure S2). Moreover, it is observed that the modified samples show increasing light absorption intensity in the range of 530–800 nm, which can be due to the plasmonic effect of newly formed Ag nanoparticles on the Ag_3_PO_4_ surfaces (Shen et al., 2018). The room-temperature PL spectra of the related samples are further shown in Figure 5B. All the samples display a strong emission peak located at around 560 nm, which can be considered as a result of the recombination of photogenerated electrons and holes of Ag_3_PO_4_ (Tian et al., 2017). It is interesting to note that the overall emission intensity of the modified samples is significantly decreased, especially for the 1P-AP sample. This indicates that the recombination of photogenerated carriers is effectively inhibited by surface modification because the newly generated Ag nanoparticles would act as electrons capture and the bounded ${\text{PO}}_{4}^{3-}$ and hydroxyl groups provide negative electrostatic filed in favoring the transfer of holes, conducing to boost photocatalytic activity.

**Figure 6:**
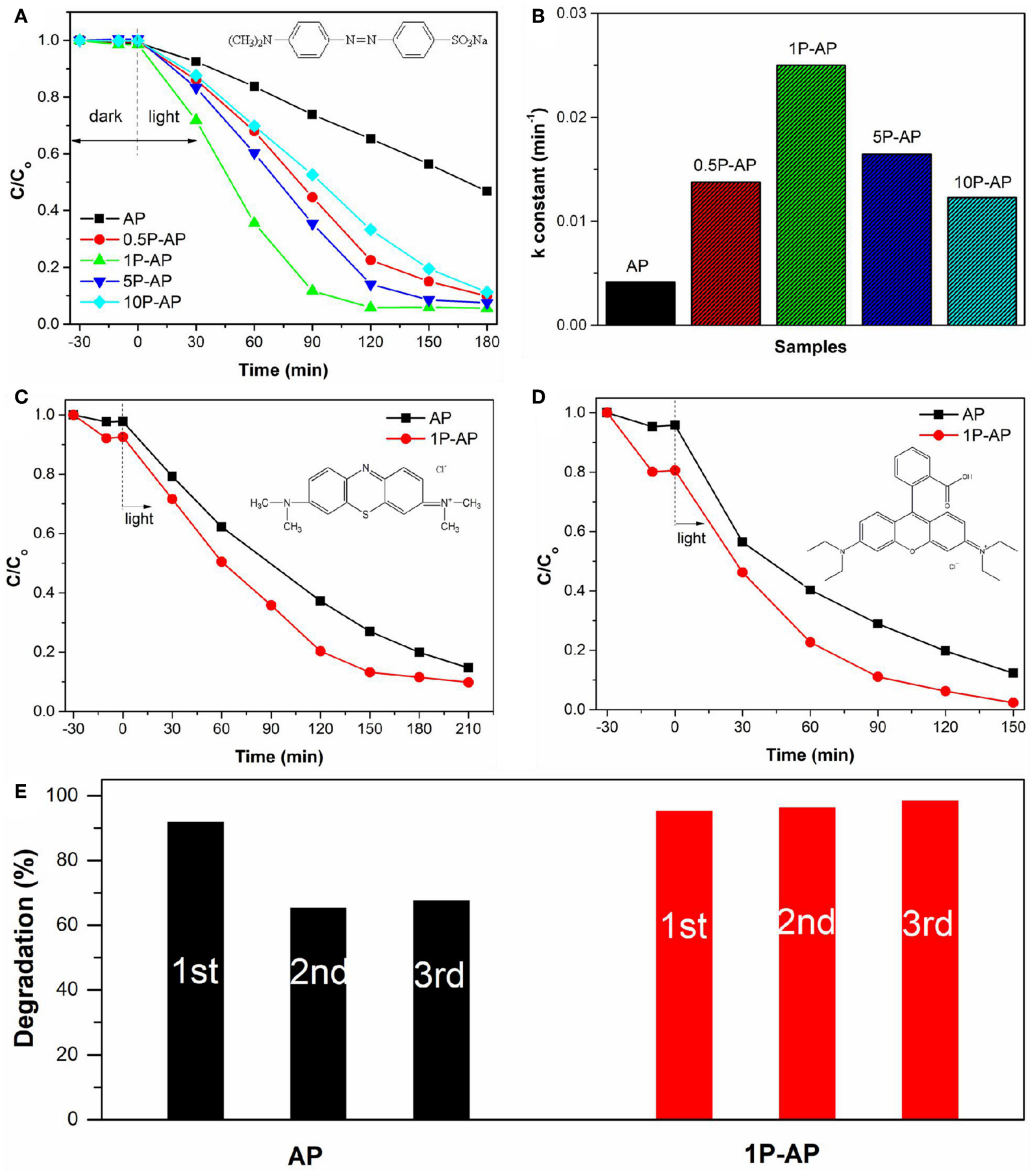
**(A)** The photocatalytic activities of bare Ag_3_PO_4_ and ammonium phosphate-modified Ag_3_PO_4_ samples toward MO degradation under visible light irradiation, and **(B)** the apparent rate constants. Photocatalytic degradation of **(C)** MB and **(D)** RhB on bare Ag_3_PO_4_ and ammonium phosphate-modified Ag_3_PO_4_ samples exposed to the visible light illumination **(E)** The photocatalytic stability of AP and 1P-AP (in 210 min of irradiation) for MB degradation.

The photocatalytic performance of bare Ag_3_PO_4_ and the modified Ag_3_PO_4_ photocatalysts were initially evaluated by decomposing MO dye which as a model pollutant in solution under visible light irradiation. As shown in Figure 6A, all the as-obtained Ag_3_PO_4_ samples before and after surface modification have almost no adsorption on the anionic dye MO, which might be because of the low BET surface area and the surface-carried negative charge of the samples. Bare Ag_3_PO_4_ (AP) could degrade MO into small molecules attributed to its high oxidation capacity; almost 35% of MO is decolorized within 120 min under light illumination. As compared, the photocatalytic performance of the modified Ag_3_PO_4_ was improved significantly and was highly dependent on the concentration of ammonium phosphate. Among them, the 1P-AP sample showed the highest activity and could degrade almost 95% of MO within the same reaction period. Moreover, it was found that the photodegradation curves of MO dye were fitted by pseudo-first-order reaction kinetics. Figure 6B gives the corresponding rate constant of various samples. Clearly, the modified samples exhibited much higher rates than the bare Ag_3_PO_4_ while the 1P-AP sample had the highest rate constant, about 5 times that of the AP sample. In view of the improved photocatalytic performance of Ag_3_PO_4_ after surface modification, we extended the test for the other two cationic dyes MB and RhB (Figures 6C,D). As expected, bare Ag_3_PO_4_ had visible adsorption for these two dyes and the modified samples exhibited enhanced adsorption because of the more negative electrostatic field provided by phosphate modification. In addition, the modified 1P-AP sample showed much higher degradation for both MB and RhB than AP, suggesting the alternative route to boost the photocatalytic performance of Ag_3_PO_4_ by surface modification.

In addition to the photocatalytic activity, the modified Ag_3_PO_4_ samples also displayed improved activity stability with respect to the bare sample. As shown in Figure 6E, after three cycles, the photocatalytic activity of AP was reduced by 30% while 1P-AP did not show any significant loss of photocatalytic activity for the degradation of MB. The excellent photocatalytic stability of 1P-AP sample can be attributed to the formation of Ag/Ag_3_PO_4_ heterostructures during the continuous photocatalytic experiments, as evidenced by the XRD and SEM characterizations of the used catalysts (Figures S3, S4). In particular, the modification of Ag_3_PO_4_ with (NH_4_)_3_PO_4_ can remarkably inhibit the decomposition of Ag_3_PO_4_ into metallic Ag, resulting in the highly active and stable Ag/Ag_3_PO_4_ heterostructures.

**Figure 7:**
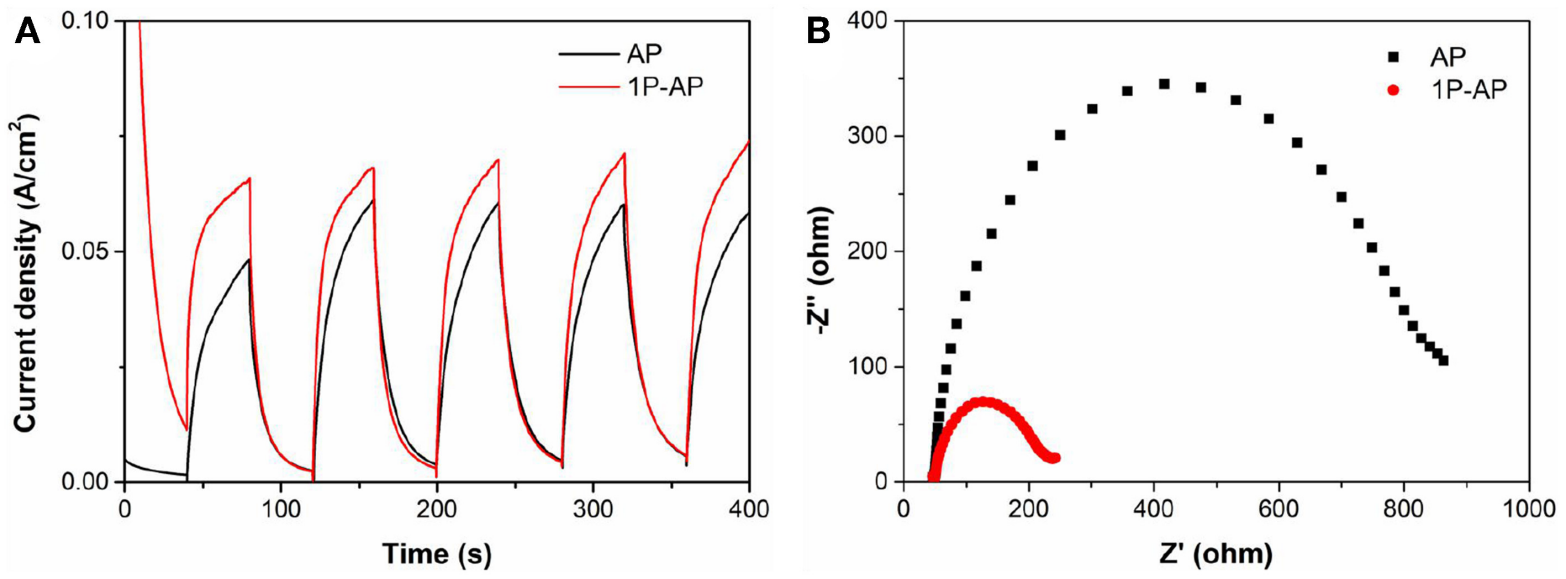
**(A)** Transient photocurrent response curves and **(B)** electrochemical impedance spectra of AP and 1P-AP.

According to what we have observed and discussed above, the enhanced photocatalytic performance of Ag_3_PO_4_ photocatalyst after surface modification can be mainly attributed to the newly formed Ag nanoparticles and the strongly bounded ${\text{PO}}_{4}^{3-}$ groups, rather than the effects of particle size and surface area. Many researchers have reported that the usually formed Ag^0^ nanoparticles distributed on the surface of Ag-based photocatalysts can function as electron acceptors to accelerate the charge segregation due to the high Schottky barrier at the interface of metal/semiconductor, inducing efficient interfacial charge transfer (Yan et al., 2014b). It is also indicated that the phosphate modification could promote the transfer of photogenerated holes to the surface of photocatalysts driven by the negative electrostatic field, leading to an improved charge separation (Xie et al., 2015). Accordingly, the synergetic effect of Ag nanoparticles and surface negative electrostatic field makes separation of charge carriers more efficient and inhibits their recombination. Thus, the transient photocurrent and electrochemical impedance tests were investigated to prove that (Figures 7A,B). As expected, the ammonium phosphate-modified sample shows a larger photocurrent and a smaller curvature radius of impedance than the bare Ag_3_PO_4_. These results are also consistent with the above-mentioned PL spectra (Figure 5B), in which the modified samples show a lower emission peak than the bare sample, and the 1P-AP sample exhibits the lowest PL emission peak.

**Figure 8:**
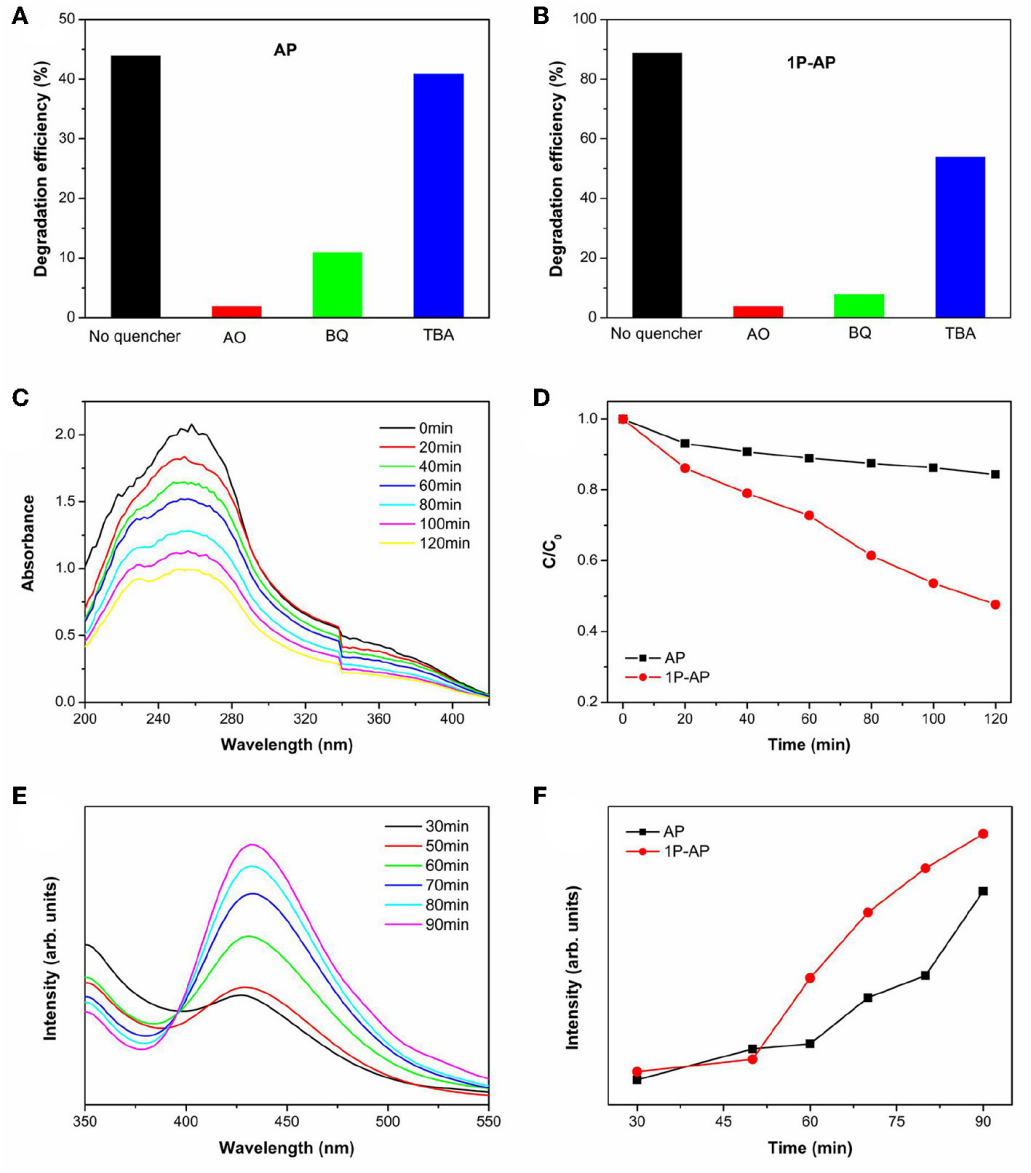
**(A)** Effects of capture on the decomposing rate of AP and **(B)** 1P-AP (in 90 min of irradiation) for MB. **(C)** UV-Vis absorption spectra of NBT under visible light irradiation in 1P-AP composite suspension, and **(D)** the plots of the induced absorbance at 249 nm with illumination time on AP and 1P-AP. **(E) ·**OH-capturing PL spectra of (λ_ex_ = 312 nm) of 1P-AP, and **(F)** the plots of the induced PL intensity at 426 nm with illumination time on AP and 1P-AP.

**Figure 9:**
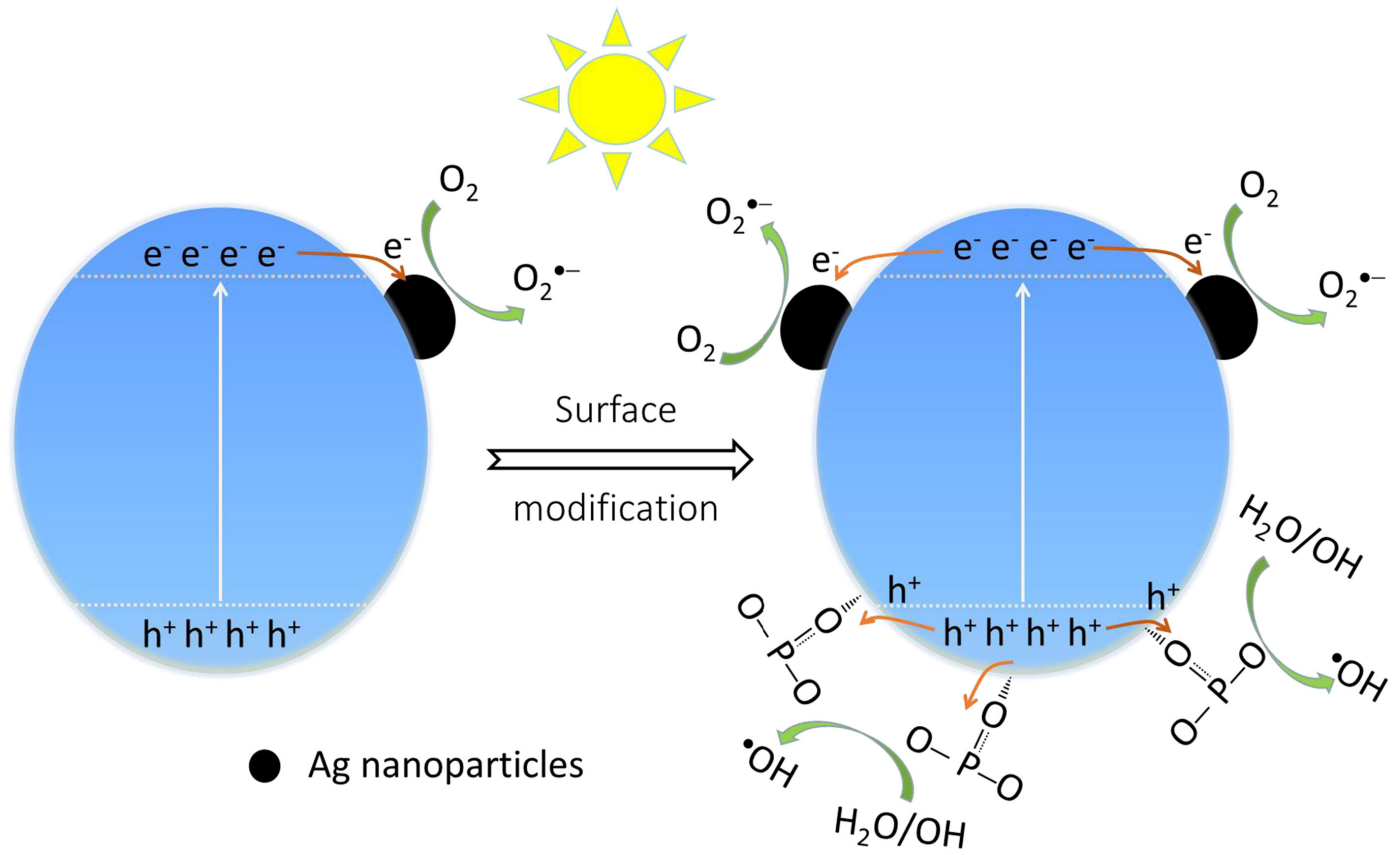
The effect of surface modification on the photocatalytic mechanism of Ag_3_PO_4_.

On the other hand, it is suggested that the surface modification by phosphate offers an attractive advantage to integrate with complex surface topologies, contribute to the forming of reactive oxygen substance (Zhong and Gamelin, 2010; Seabold and Choi, 2011; Zhong et al., 2011). As a consequence, it is necessary to probe the main active substance during photocatalysis on the Ag_3_PO_4_ photocatalysts to disclose the improved photocatalytic performance. We can observe that the decomposition efficiency of MB over bare Ag_3_PO_4_ after adding various scavengers (Figure 8A). The addition of benzoquinone (BQ) and oxalic acid ammonia (AO) significantly restrained the photocatalytic performance of bare Ag_3_PO_4_, which indicates that ${\text{O}}_{2}^{\cdot \text{−}}$ and h^+^ are the main active species over bare Ag_3_PO_4_ in photocatalysis, consistent with the reported results (Yan et al., 2014b; Zhai et al., 2016). As compared, it is observed from Figure 8B that the degradation of MB by the modified Ag_3_PO_4_ after BQ and AO addition is also significantly restrained, indicating that ${\text{O}}_{2}^{\cdot \text{−}}$ and h^+^ are also the main active substances of modified Ag_3_PO_4_; meanwhile, the degradation activity was also inhibited after the addition of tertbutyl alcohol (TBA), indicating that **·**OH can also serve as active species in 1P-AP. It should be noticed that the 1P-AP sample suffered more suppression by the addition of BQ than AP, suggesting the more contribution of ${\text{O}}_{2}^{\cdot \text{−}}$ in 1P-AP by the increased amount of metallic Ag nanoparticle. The presence of ${\text{O}}_{2}^{\cdot \text{−}}$ radicals can be confirmed by a nitroblue tetrazolium (NBT) probe method (Yan et al., 2015). As shown in Figure 8C, the characteristic peak at 259 nm shows a gradual decrease in intensity with prolonging irradiation time, suggesting the reaction between NBT and ${\text{O}}_{2}^{\cdot \text{−}}$ radicals, indirectly evidencing the presence of ${\text{O}}_{2}^{\cdot \text{−}}$ radicals. In particular, under the same conditions, 1P-AP can produce more ${\text{O}}_{2}^{\cdot \text{−}}$ radicals than AP sample (Figure 8D), coinciding with the activity trend and the results of BQ quenching. Moreover, driven by the strong bound ability of ${\text{PO}}_{4}^{3-}$ and the induced negative electrostatic field, photogenerated holes would migrate to the surface of 1P-AP and react with hydroxyl groups to form **·**OH. The increased reactive **·**OH can be further supported by the measure of photoluminescence technique with terephthalic acid (PL–TA). The results in Figure 8E indicates that a significant PL emission peak located at around 426 nm is observed, which is monotonously increased against the irradiation time, evidencing the formation of **·**OH radicals in the photocatalysis. As shown in Figure 8F, under the same irradiation time, the 1P-AP sample displays much higher PL intensity than AP, demonstrating the high amount of generated **·**OH radicals on the modified photocatalyst. As a consequence, surface modification of Ag_3_PO_4_ favors the efficient charge of photogenerated electrons and holes to Ag nanoparticles and bounded OH groups, respectively, facilitating more charge carriers to produce various reactive oxygen substance and participation in the photocatalysis (Figure 9).

The photocatalytic activity of modified Ag_3_PO_4_ is also dependent on the concentration of ammonium phosphate, which has an obvious effect on the size and distribution of Ag nanoparticles as well as the anion groups bounded on the Ag_3_PO_4_ surfaces. For example, at a moderate concentration (1 mM), the nearly monodispersed Ag nanoparticles with small size construct a good heterostructure interface with Ag_3_PO_4_, accelerating the transfer of electron–hole pairs and producing highly reactive ${\text{O}}_{2}^{\cdot \text{−}}$; while the surface modification at high concentration (10 mM) would induce more Ag nanoparticles but they are highly aggregated and grow into larger particles, therefore contribute to a negative effect on the photocatalytic activity. At the same time, the more adsorbed ${\text{PO}}_{4}^{3-}$ would also substitute the surface OH groups, possibly reducing the chance of direct holes reacting with surface hydroxyl groups to generate reactive **·**OH radicals.

To further distinguish the single role of ${\text{NH}}_{4}^{+}$ and ${\text{PO}}_{4}^{3-}$ in promotion of photocatalytic performance of Ag_3_PO_4_, two control catalysts that were modified with NH_4_NO_3_ and Na_3_PO_4_, and followed by the thermal treatment process were prepared and denoted as 1NH_4_NO_3_-AP and 1Na_3_PO_4_-AP, respectively. As shown from the XRD results (Figure S5), the (210) peak of 1NH_4_NO_3_-AP displays a left shift as compared with bare AP, indicating the separation of silver ions from the crystal lattice driven by the strong coordination of ${\text{NH}}_{4}^{+}$, in good agreement with the results of ammonium etching (Zhai et al., 2016). In the case of 1Na_3_PO_4_-AP, there is no obvious change in the XRD peaks as compared with bare AP, but the photocatalytic activity increased obviously (Figure S6), which indicates the positive effect of ${\text{PO}}_{4}^{3-}$. Moreover, we can observe that the activity trend follows the order of 1(NH_4_)_3_PO_4_-AP > 1Na_3_PO_4_-AP ≈ 1NH_4_NO_3_-AP > bare Ag_3_PO_4_, strongly supporting the multiple roles of ammonium phosphate in promotion the photocatalytic performance of Ag_3_PO_4_.

## Conclusion

We have developed a one-pot surface modification route by using ammonium phosphate solutions to improve the photocatalytic performance of Ag_3_PO_4_. It was found that ammonium phosphate plays the multiple promotion roles in favoring the formation of metallic Ag nanoparticles and providing the negative electrostatic field on the surface of Ag_3_PO_4_ photocatalysts, which consequently promote the separation efficiency of photoinduced electron-hole pairs, enhance the selective adsorption of cationic dye, and increase the concentration of reactive oxygen species. This work provides an alternative route to boost the photocatalytic activity of Ag_3_PO_4_ and can spread to design and fabricate other potential Ag-based photocatalytic materials.

## Data Availability Statement

The datasets generated for this study are available on request to the corresponding author.

## Author Contributions

QL and ZQ conducted the catalysts preparation. QL, LW, and SZ performed the activity test. NL, ZJ, and WL discussed the mechanism part. QL, NL, and TY conceived the project and co-wrote the manuscript. The manuscript was written through collective contributions from all authors. All authors approved the final version of the manuscript.

### Conflict of Interest

The authors declare that the research was conducted in the absence of any commercial or financial relationships that could be construed as a potential conflict of interest.
