# Supplementary material for: The Multiple Promotion Effects of Ammonium Phosphate-Modified Ag3PO4 on Photocatalytic Performance
Source: Front Chem. 2019 Dec 24;7:866. doi: 10.3389/fchem.2019.00866 (PMC6937216; doi:10.3389/fchem.2019.00866)
Supplement: Supplementary file 1 [file Data_Sheet_1.pdf]

The multiple promotion effects of ammonium phosphate-modified Ag_3PO_4 on photocatalytic performance

Qin Liu[1], Na Li[2]*, Zheng Qiao[1], Wenjuan Li[1], Linlin Wang[1], Shuao Zhu[1], Zhihong Jing[1], Tingjiang Yan[1]*

[1]The Key Laboratory of Life-Organic Analysis, College of Chemistry and Chemical Engineering,
[2]Qufu Normal University Library, Qufu Normal University, Qufu, Shandong 273165, P. R. China

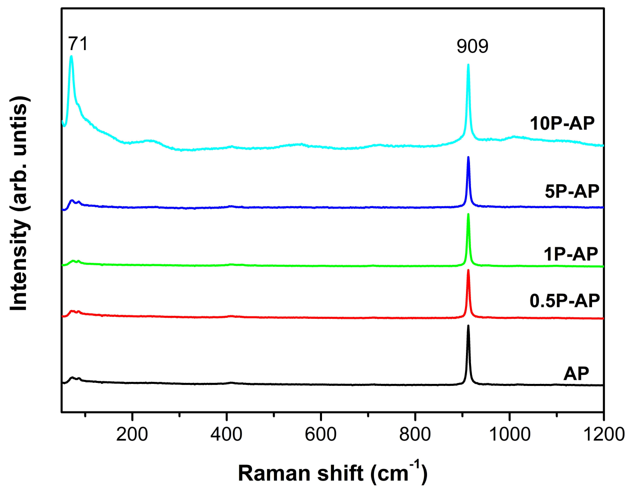

Fig. S1 Raman spectra of bare Ag_3PO_4 and ammonium phosphate-modified Ag_3PO_4 samples.

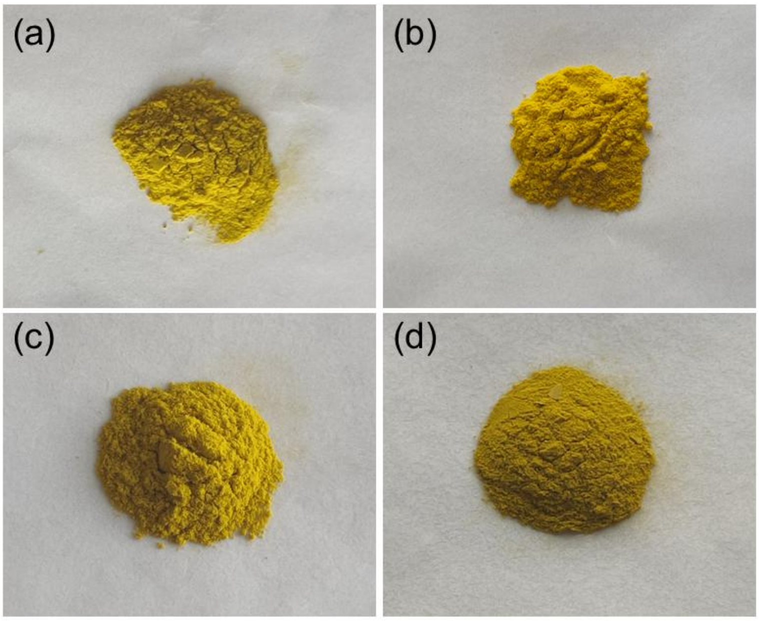

Fig. 2 The color of bare Ag_3PO_4 (**a**) and ammonium phosphate-modified Ag_3PO_4 samples: 0.5P-AP (**b**), 1P-AP (**c**), and 10P-AP (**d**).

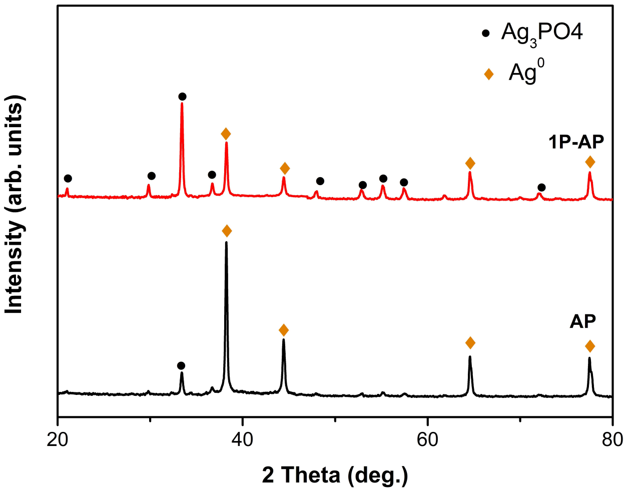

Fig. S3 The XRD patterns of bare Ag_3PO_4 (AP) and ammonium phosphate-modified Ag_3PO_4 (1P-AP) after photocatalytic experiments.

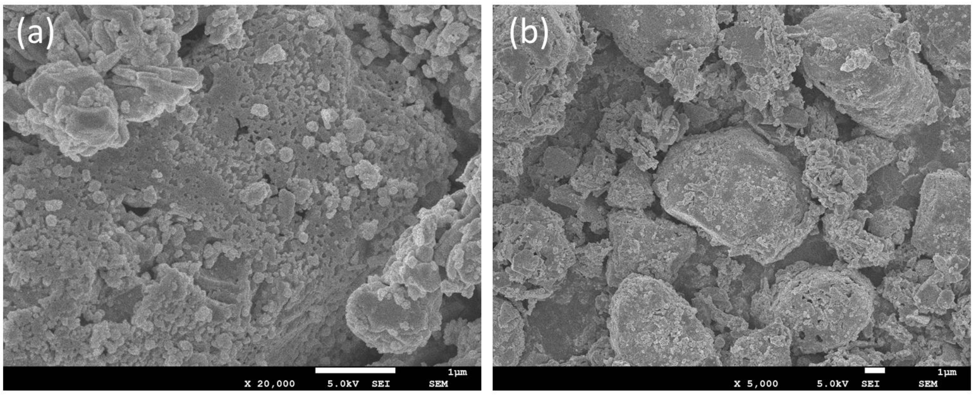

Fig. S4 The SEM of bare Ag_3PO_4 (AP) (**a**) and ammonium phosphate-modified Ag_3PO_4 (1P-AP) (**b**) after photocatalytic experiments.

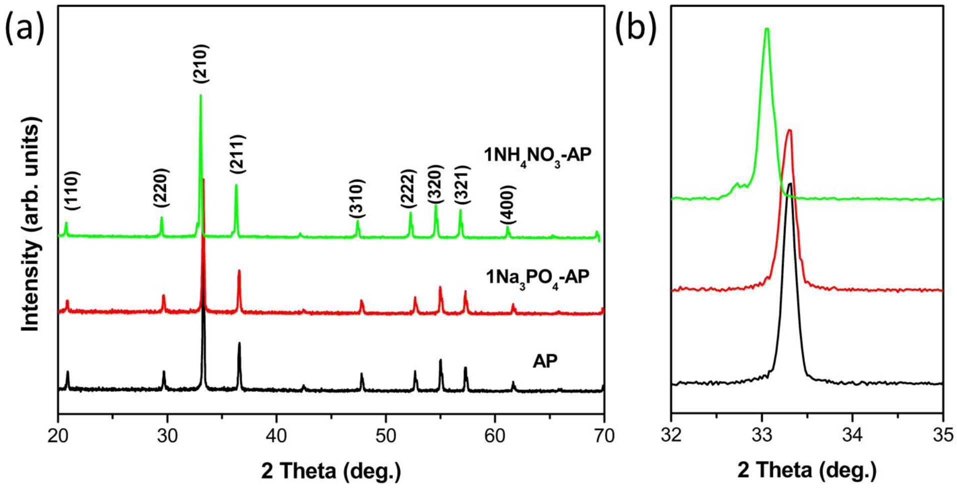

Fig. S5 (**a**) XRD patterns of bare Ag_3PO_4 (AP) and modified Ag_3PO_4 samples ($1Na_3PO_4$-AP, $1NH_4NO_3$-AP), (**b**) enlarged XRD patterns from 32 ^{o} to 35 ^{o}.

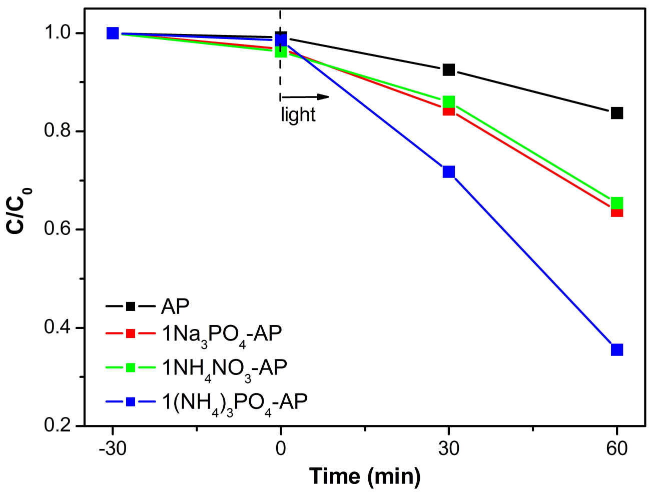

Fig. S6 The photocatalytic activities of bare Ag_3PO_4 (AP), $1Na_3PO_4$-AP, $1NH_4NO_3$-AP and $1(NH_4)_3PO_4$-AP toward MO degradation under visible light irradiation.

Table S1 Surface area, pore size and pore volume parameters for bare Ag_3PO_4 (AP) and ammonium phosphate-modified Ag_3PO_4 (1P-AP).

**Photocatalysts**	**S_{BET} (m^2 g^{-1})**	**Pore diameter (nm)**	**Pore volume (cm^3 g^{-1})**
AP	3.6045	3.3469	0.003014
1P-AP	2.9716	2.96625	0.002204

Table S2 The ratio of $(NH_4)_3PO_4$ in the 10P-AP composites obtained from XPS results.

**Sample**		**10P-AP**		
**Elements**	Ag	O	P	N
**Atomic %**	36.59	45.79	13.17	4.45
**Substance**	Ag^0	Ag_3PO_4		$(NH_4)_3PO_4$
**Ratio %**	10.34	79.6		10.1
